# Metabolic Disturbance and Th17/Treg Imbalance Are Associated With Progression of Gingivitis

**DOI:** 10.3389/fimmu.2021.670178

**Published:** 2021-06-21

**Authors:** Weijie Wang, Xinchang Wang, Shuhao Lu, Huiqing Lv, Ting Zhao, Guanqun Xie, Yu Du, Yongsheng Fan, Li Xu

**Affiliations:** ^1^ Department of Rheumatology, The Second Affiliated Hospital of Zhejiang Chinese Medical University, Hangzhou, China; ^2^ School of Basic Medical Science, Zhejiang Chinese Medical University, Hangzhou, China; ^3^ School of Pharmaceutical Sciences, Zhejiang Chinese Medical University, Hangzhou, China

**Keywords:** metabolic disturbance, gingivitis, Treg, Th17, metabolism

## Abstract

**Objective:**

This study sought to explore the role of metabolic disturbance in immunoregulation of gingivitis targeting T helper 17 cells (Th17)/regulatory T cell (Treg).

**Materials and Methods:**

A total of 20 gingivitis patients and 19 healthy volunteers were recruited. Quantitative real time polymerase chain reaction (qRT-PCR) was used to evaluate expression patterns of Forkhead box protein P3 (Foxp3), transforming growth factor-β (TGF-β), retinoid-related orphan receptor-gammat (RORγt) and interleukin 17A (IL-17A) in the peripheral blood lymphocytes of subjects across the two groups. Moreover, the enzyme-linked immunosorbent assay (ELISA) technique was used to detect levels of TGF-β, IL-4, IL-6,TL-10 and L-17A secreted in the plasma as well as the SIgA secreted in saliva. Flow cytometry was used to detect the percentage of CD4^+^CD25^+^ Foxp3^+^Treg cells and the percentage of CD4^+^IL-17A^+^ Th17 cells in whole blood of subjects in both groups. Gas chromatography-mass spectrometry (GC-MS) was employed to analyze the plasma metabolites in the gingivitis patient group. Statistical analysis was applied to determine whether the plasma metabolites and related metabolic pathways significantly differed between gingivitis patients and healthy controls. Ingenuity pathway analysis (IPA) was employed to identify the potential relation between the metabolites and the Th17 and Treg related pathway.

**Results:**

The percentages of CD4^+^IL17A^+^Th17 cells and IL-17 significantly increased in the peripheral blood in the gingivitis group. Moreover, the upregulation of IL-17A mRNA and RORγt mRNA were also found in the gingivitis group. However, the percentage of CD4^+^CD25^+^ Foxp3^+^Treg cells and Foxp3 mRNA in the whole blood did not significantly change. However, TGF-β mRNA as well as TGF-β, IL-4, IL-6, IL-10 in the periperial blood and SIgA in the saliva were higher in the gingivitis group. Notably, that the ratio of Th17/Treg cells was significantly increased during peripheral circulation. Furthermore, we identified 18 different metabolites which were differentially expressed in plasma between the gingivitis and healthy control groups. Notably, the levels of cholesterol, glycerol 1-octadecanoate, d-glucose, uric acid, cyclohexaneacetic acid, 3-pyridine, tryptophan, and undecane 2,4-dimethyl were significantly up-regulated. whereas the levels of lactic acid, glycine, linoleic acid, monopalmitic acid, glycerol, palmitic acid, pyruvate, 1-(3-methylbutyl)-2,3,4,6-tetramethylbenzene, 1 5-anhydro d-altrol, and boric acid were down-regulated in the gingivitis group, relative to healthy controls. IPA showed that these metabolites are connected to IL17 signaling, TGF-B signaling, and IL10 signaling, which are related closely to Th17 and Treg pathway.

**Conclusion:**

Overall, these results showed that disturbance to glycolysis as well as amino and fatty acid metabolism are associated with Th17/Treg balance in gingivitis. Impaired immunometabolism may influence some periodontally involved systemic diseases, hence it is a promising strategy in targeted development of treatment therapies.

## Introduction

Epidemiological studies have estimated that more than 82% of juveniles in the United States exhibit gingivitis and gingival bleeding (1). In China, the National Oral health Epidemiological Survey report revealed that currently, about 80 - 97% of adults exhibit different degrees of periodontal disease (2). Gingivitis can induce gingival diseases such as periodontitis, which is the main cause of loss of teeth in Chinese adults. This gum disease can exacerbate the risk of various systemic diseases, such as diabetes, rheumatoid arthritis, and inflammatory bowel disease (3, 4).

Periodontitis is a chronic infectious disease that starts as an inflammation of the periodontal tissues, eventually causing resorption of alveolar bone and subsequent loss of teeth. The disease is characterized by inflammation and bleeding of the gums, periodontal pocket formation, and gradual loss of attachment. Dental plaque biofilm is a major etiological factor associated with the development of periodontitis. Generally, periodontal pathogens enter periodontal tissues and intentionally or unintentionally cause differentiation of osteoclasts as well as absorption of alveolar bone, thereby facilitating development of periodontitis, and activating intrinsic cells. Consequently, the activated T and B cells initiate adaptive immunity by antigen-presenting cells after 21 days of innate immunity. Previous studies have demonstrated that conversion of gum tissue lesions into periodontitis is mediated by T cells (5).

Interestingly, recent studies have shown that gingivitis and periodontitis are correlated with imbalance of Th17/Treg-related cytokines (6). Notably, Th17 cells have been found to play a detrimental role in balancing of periodontitis immunity, although dysregulation of Tregs in atherosclerosis has also been reported. Multiple reports have suggested that autoimmune response induced by Th17/Treg imbalance may be one of the significant factors causing periodontitis (7, 8). Furthermore, exosomes from PDLSCs have been implicated in alleviation inflammatory microenvironment through Th17/Treg/miR‐155‐5p/SIRT1 regulatory network causing periodontitis (9). Similarly, Gao et al. (10)opined that the difference between Treg and Th17 also showed significant changes with different degrees of chronic periodontitis. In this context, unraveling the role of pathogenesis of Th17/Treg imbalance during development of gingivitis is crucial to development of treatment therapies.

To date, immune cells are considered highly dynamic with regards to their growth, proliferation, and effector functions during response to immunological challenges. In fact, different immune cells may adopt different metabolic configurations that enable the cell to balance its energy, molecular biosynthesis, and longevity requirements (11). Previous studies have reported a significant elevation of many metabolites associated with inflammation, oxidative stress, tissue degradation, and bacterial metabolism in periodontal disease (12). Moreover, previous evidences have also established that manipulating glycolytic versus oxidative metabolism influences development of long-lived memory T cells. On the other hand, inhibiting glycolysis enhances formation of memory T cells, while blocking synthesis of oxidation-dependent fatty acid OxPhos represses formation of memory T cells (13, 14). Based on this, we sought to elucidate the role of Th17/Treg imbalance and immune metabolism in development of gingivitis.

## Materials And Methods

### Study Design

This observational study enrolled 20 gingivitis patients and 19 healthy volunteers at the Zhejiang Chinese Medical University and the Second Affiliated Hospital of Zhejiang Chinese Medical University between December 2016 and December 2017. The study protocol was approved by the Ethics Committees of Zhejiang Chinese Medical University (No.2014zjtcm-001). All participants provided informed consent prior to recruitment in the study.

### Inclusion Criteria

Participants were eligible if they were aged between 18 and 60 years, had at least 20 teeth with a clinical sign of generalized plaque-induced gingivitis, and signed a written informed consent form.

### Exclusion Criteria

Participants were excluded from the study if they: (a) were or planned to get, or were at risk of becoming pregnant without contraception; (b) were diagnosed with tumors, severe disease, immune, mental, and other diseases of the target organs of each system; (c) had undergone orthodontic surgery or tooth extraction three months prior to start of the study; (d) were taking antibiotics; and (d) had a history of drug abuse.

### Blood Collection

Five ml of venous blood was drawn from subjects, on an empty stomach, between 6:00 -8:00 in the morning. 1 ml of the blood was collected in a tube with heparin sodium anticoagulant and used for flow detection, while the remaining 4ml was mixed with ethylene diamine tetra-acetic acid (EDTA) and used to separate plasma and peripheral blood mononuclear cells (PBMC) by Lymphocyte Separation Medium (Multisciences, Hangzhou, China) according to the manufacturer’s instructions. In brief, 4 ml of lymphocyte separation solution was added into an appropriate centrifuge tube, under aseptic conditions, mixed with an equal amount of PBS, and the mixture carefully added above the level of the lymphocyte separation fluid. The contents were centrifuged at 1500 rpm, for 15 minutes at room temperature. The centrifuge tube was taken out, and the mixture divided into four layers from top to bottom, with the first, second, third and fourth layers representing plasma, PBMC, transparent separation liquid layer, and red blood cells, respectively. The plasma and PBMC were divided into several microfuge tubes (1.5 ml each) and stored at -80°C until further experiments. We also recorded the name, gender, age, weight, and severity of gingivitis

### Saliva Collection

Participants were asked to gently rinse their mouth after half an hour, and 2 ml of saliva collected into sterile containers. The saliva was aliquoted into 1.5 ml microfuge tubes and stored at - 80°C within one hour of collection for later use.

### RNA Isolation and Quantitative Real Time Polymerase Chain Reaction

Total RNA was isolated from PBMCs using the TRIzol reagent (Invitrogen, Carlsbad, CA, USA), according to the manufacturer’s instructions, and its concentration determined using a NanoDrop spectrophotometer (Thermofisher,USA). 500 ng of the total RNA was reverse-transcribed into complementary DNA (cDNA) using the PrimeScript RT Master Mix kit (Perfect Real Time, Takara, Japan), then used for quantitative real time polymerase chain reaction (qRT-PCR) analysis using the LightCycler 480 SYBR Green kit (Roche Applied Science, Penzberg, Germany). Amplification was done targeting the TGF-β, Foxp3, RORyt and IL-17A genes. Primer sequences for these genes are outlined in **Table 1**. Each sample was analyzed in triplicate, with GAPDH also included as an internal amplification control gene for normalization. Relative gene expression was calculated using the 2^-△△CT^ method.

**Table 1:** Primer sequences for genes targeted in qRT-PCR.

Gene	Forward primer (5’–3’)	Reverse primer (5’–3’)	bp
TGF-β	TGACAAGTTCAAGCAGAGTA	TGAGGTATCGCCAGGAAT	185
Foxp3	CCAGAGTTCCTCCACAAC	TGTTCGTCCATCCTCCTT	366
IL-17A	AAGACTGAACACCGACTAAG	CTCCAAAGGAAGCCTGA	228
RORC	TCAAAGCAGGAGCAATGGAAGT	GGGAGTGGGAGAAGTCAAAGATG	156
GAPDH	GGAAGCTTGTCATCAATGGAAATC	TGATGACCCTTTTGGCTCCC	168

### Enzyme-Linked Immunosorbent Assay (ELISA)

Levels of TGF-β and IL-17A in plasma as well as SIgA in saliva were determined as described in manufacturers’ protocols of the Human TGF-β ELISA Assay kit (Shanghai Yuanye Biotechnology, China), Human IL-17A ELISA Assay kit (Shanghai Yuanye Biotechnology, China) and Human SIgA ELISA Assay kit (Shanghai Yuanye Biotechnology, China), respectively, then compared between gingivitis patients and healthy controls Optical density (OD) for each sample was determined at 450 nm wavelength within 15 min using a multifunctional enzyme plate analyzer. We used the standard concentration as the abscissa and the corresponding OD value as the ordinate to draw a standard linear regression curve, then calculated concentrations value of each sample according to the curve equation.

### Flow Cytometry

Fresh heparinized peripheral blood samples were collected from each group. 200 µl of whole blood sample was collected in a flow tube, based on pre-test and reagent instructions, and subjected to cell staining. The contents were incubated with 10 µl anti-human CD4 (FITC, clone OKT4, Catalog # 11-0048-42,eBioscience, USA) and 10 µl anti-human CD25 (APC, clone BC96, Catalog #17-0259-42, eBioscience, USA) for 30 min at room temperature.Later, 2 ml 1×FCM Lysing Solution (Catalog #70-LYS01,Multiscienses, China) was added to each tube and incubated at room temperature for 15 min in the dark. The product was centrifuged at 350g for 5 min, and the supernatant was discarded. Then 2 ml PBS was used for washing 3 times. Next,1 microliter 1× Intracellular Fixation & Permeabilization Buffer (Catalog # 88-8823-88 eBioscience, USA) was added at room temperature in the dark for 60 min. Thereafter, the contents were mixed with 1 ml 1×Permeabilization Buffer (Catalog #00-8333-56 eBioscience, USA), centrifuged at 350 g at 4°C for 6 minutes, the cells resuspend in 100 µl 1×Permeabilization Buffer. 15 µl of anti-human Foxp3 (PE, clone PCH101, Catalog # 12-4776-42, eBioscience, USA) and rat IgG2a K Isotype Control (PE, clone eBR2a, Catalog # 12-4321-41,eBioscience, USA) were added to the tube, followed by a 30-min incubation at 37°C in the dark. An additional 2 ml 1×Permeabilization Buffer was added to the contents, followed by centrifugation for 6 min at 350 g at 4°C. Finally, the cells were resuspended in 500 µl Flow Cytometry Staining Buffer (Catalog # 00-4222-57, eBioscience, USA) and subjected to flow cytometry on the flow cytometer (BECKMAN,USA). A total of 5×10^4^ events were acquired for each sample *via* flow cytometry.

Th17 cells were also detected by flow cytometry. Briefly, 250 μl heparin anticoagulated whole blood was inoculated into RPMI-1640 cell culture media, supplemented with 10% fetal bovine serum and 1% double-antibody, and cultured in a 24-well plate at 37°C in a 5% CO_2_. Thereafter, 1.5 μl of Leukocyte Activation Cocktail with BD GolgiPlug™ (Catalog #550583, BD Biosciences, USA) was added to each well, and incubated for 4.5 h. The cultures were incubated with anti-human CD4 (FITC, clone RPA-T4, Catalog # 55346, BD Biosciences, USA) for 15 min at room temperature in the dark. And then,100µl MEDIUM A of FIX&PERM Kit(Catalog # 70-GAS003,Multisciences,China) was added and incubated at room temperature for 15 min. Three ml Flow cytometry Staining buffer was used for washing. Later, 100µl MEDIUMB of FIX&PERM Kit was added and mixed. After fixation and rupture, cells were incubated with anti-human IL-17A (PE, clone N49-653, Catalog #560486, BD Biosciences, USA) for 15 min at room temperature in the dark. Three ml Flow cytometry Staining buffer was used for washing. Finally, the cells were resuspended in 500 µl Flow Cytometry Staining Buffer and subjected to flow cytometry on the flow cytometer. Non-stimulation of the Leukocyte Activation Cocktail of the Th17 cells from one healthy control subject was used for biological control. Data were analyzed using the FlowJo software 10.0 (Flowjo, Ashland, OR).

### Sample Preparation for Metabolic Profiling Analysis

Plasma samples were first thawed and homogenized at room temperature, Then 200 μL cold methanol with an internal norm applied to 50 μL of plasma for protein precipitation, in an ice-water bath. The contents were mixed by vortexing, for 30 s, centrifuged at 12,000 g at 4°C for 10 min, the supernatant transferred to microfuge tubes and freeze-dried using the Labconco CentriVap system (Kansas, MO, USA. The lyophilized residue was oxymated by adding 50 μL of methoxyamine pyridine solution (20 mg/mL) and incubated for 90 min in a water bath at 40°C. Thereafter, 40 μL of MSTFA was added and incubated for 60 min for trimethylsilylation. To verify the repeatability and stability of the sample analysis, for every 10 real samples, quality control (QC) samples were added to the analysis chain. The QC sample was prepared by combining the plasma samples tested similarly, with these samples collected and analyzed alongside the real samples.

### Gas Chromatography-Mass Spectrometry (GC–MS) Analysis Conditions

GC–MS analysis was performed on an Agilent 7890/5975C GC–MS (Agilent Technologies, Santa Clara, CA, USA), with chromatographic separation achieved using a DB-5 fused silica capillary column (30 m × 0.25 mm × 0.25 μm) (J&W Scientific, Folsom, CA, USA). The flow rate of the carrier gas (high-purity helium, 99.9996%) was 1.2 mL/min, and the column oven heating was programmed as follows: after initial holding at 70°C for 3 min, the temperature was increased to 300°C at a rate of 5°C/min, and kept it maintained there for 5 min. The injection volume and split ratios were 1 μL and 5:1, respectively, while the injection port and transfer line temperatures were 300 and 280°C, respectively. The ion source (EI) temperature was 230°C, whereas the mass spectrum scan range and solvent delay time were m/z 33~600 and 4.8 min, respectively. All samples were analyzed in a random order, and QC samples were injected within the sequence.

### Data Processing and Data Analysis

Raw data from subjects in both groups were analyzed using the Mass Profiler Professional (Agilent) software. The data were normalized and post-edited using Microsoft EXCEL 2010 software, and final results organized into a two-dimensional data matrix, including variables (rt_mz, retention time_mass-to-charge ratio), molecular weight (mass), observations (sample), and peak intensity. The edited data matrices were imported into SIMCA-P software (version 13.0) and subjected to unsupervised principal component analysis (PCA) as well as supervised partial least squares discriminant analysis (PLS-DA) for pattern recognition. We adopted the supervised method orthogonal partial least-squares discriminant analysis (OPLS-DA) for modeling to distinguish between the gingivitis and control groups. To identify differentially expressed metabolites, we applied the variable importance in projection (VIP) value (threshold value > 1) of the OPLS-DA model, coupled with the *P*-value of the t-test (*P* < 0.05). Qualitative differential analysis of metabolites was performed using the following method: search an online database (Metlin) (compare mass-to-charge ratio m/z or accurate molecular mass) and input the total difference substances obtained into MetaboAnalyst 3.0 to obtain the metabolic pathways where these different substances are located.

### Identification of Potential Biomarkers

To identify significantly changed metabolites, we searched the National Institute of Standards and Technology mass spectral library (NIST05, Gaithersburg, MD, USA) for similar metabolites. Subsequently, we used the NIST Mass Spectral Search Program Version 2.0 (http://www.nist.gov/srd/mslist.htm) to match the molecular formula and structure of the identified compounds. Finally, we performed metabolic pathway analysis of the potential biomarkers using the KEGG (http://www.kegg.com/) and HMDB (http://www.hmdb.ca/) databases.

### Ingenuity Pathway Analysis (IPA)

IPA is an all-in-one integrated online analytics software, entirely based on the life sciences data collected by the Ingenuity Knowledge Base. Functionally, this software helps to reveal properties of various molecules, such as biomarkers related to pathways of genes, proteins, chemicals and active ingredients of drugs, as well as network interactions among them. The identified differential biomarkers, along with their attributes and corresponding variables were analyzed IPA software, which allowed effective and intuitive acquisition of potential targets and pathways in the gingivitis group relative to healthy controls.

### Statistical Analysis

Statistical analyses were performed using SPSS software, version 20.0. Normally distributed data were expressed as means ± standard deviation (SD), while non-normal data were presented as medians with their interquartile ranges. For uniform variances, an independent-sample *t*-test was used for comparison between the two groups, while for non-uniform variances, a non-parametric test was applied. Data processing and graphing were performed using GraphPad Prism 5 software, and data followed by *P* < 0.05 considered statistically significant.

## Results

### Baseline Characteristics of Participants

**Figure 1:**
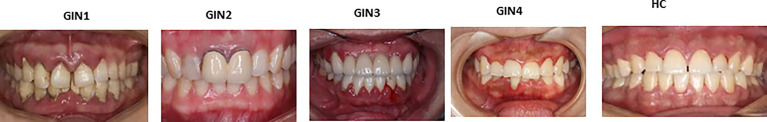
Clinical manifestations of gingivitis participants and healthy controls.

Thirteen and seven cases in the gingivitis group were female and male, respectively, whereas the proportion of males to females in the healthy control group was equal. Participants in both groups were aged between 25 and 50 years, and their body mass indices (BMI) were within the normal range. Gingivitis patients had significantly higher Sulcus bleeding index (SBI) and gingival index (GI) than their healthy counterparts (*P* < 0.001). The patients’ gingival was swollen and sore (**Figure 1**), suggesting local inflammation. Demographic and clinical chemistry characteristics of the two groups are summarized in **Table 2**. Specifically, there were no significant differences in gender and age between the gingivitis and control groups (P > 0.05). However, white blood cell (WBC) counts and percentage neutrophils were significantly higher in the gingivitis patients, relative to healthy controls. Conversely, Lymphocytes percentage were significantly lower in the gingivitis, than in the control, group. These results indicated that immune system disorder is activated upon gingivitis development.

**Table 2:** Demographic and clinical chemistry characteristics of the participants.

	HC	GIN
number	19	20
years	36.88 ± 11.28	38.46 ± 12
Male/Female	11/8	13/7
BMI	20.03 ± 1.36	23.15 ± 3.61
SBI	0.67 ± 0.18	1.27 ± 0.2***
GI	0.65 ± 0.17	1.20 ± 0.17***
WBC(10^9/L)	6.00 ± 0.92	7.77 ± 2.61*
RBC(10^12/L)	4.95 ± 0.46	5.12 ± 0.45
Hemoglobin(g/L)	147.32 ± 18.79	153.32 ± 11.15
PLT(10^9/L)	226.08 ± 40.21	236.56 ± 48.28
Neutrophils percentage(%)	52.12 ± 7.86	64.85 ± 8.93**
Lymphocytes percentage(%)	36.42 ± 7.55	25.81 ± 7.65**
Monocytes percentage(%)	7.32 ± 1.59	7.13 ± 1.57
Acidophil percentage(%)	2.66 ± 1.73	2.89 ± 2.24
Basophils percentage(%)	0.48 ± 0.18	0.37 ± 0.14
Glucose(mmol/L)	4.67 ± 0.36	5.01 ± 0.65
Uric acid(μmol/L)	339.68 ± 78.8	372.35 ± 60.72
Creatinine(μmol/L)	55.42 ± 31.02	48.6 ± 38.05
Triglyceride(mmol/L)	1.32 ± 0.55	1.48 ± 0.67
ALT(U/L)	24.39 ± 19.05	24.45 ± 14.48
AST(U/L)	24.42 ± 17.02	24.53 ± 15.28

Data presented are means ± SD. *P < 0.05, **P < 0.01, ***P < 0.001, compared to HC group. BMI, body mass index; SBI, Sulcus bleeding index; GI, gingival index; WBC, white blood cell; RBC, red blood cell; PLT, platelet; ALT, alanine aminotransferase; AST, aspartate aminotransferase. GIN, the gingivitis group; HC, the healthy control group.

### Proportion and Ratio of Th17/Treg in Peripheral Blood of Gingivitis Patients

**Figure 2:**
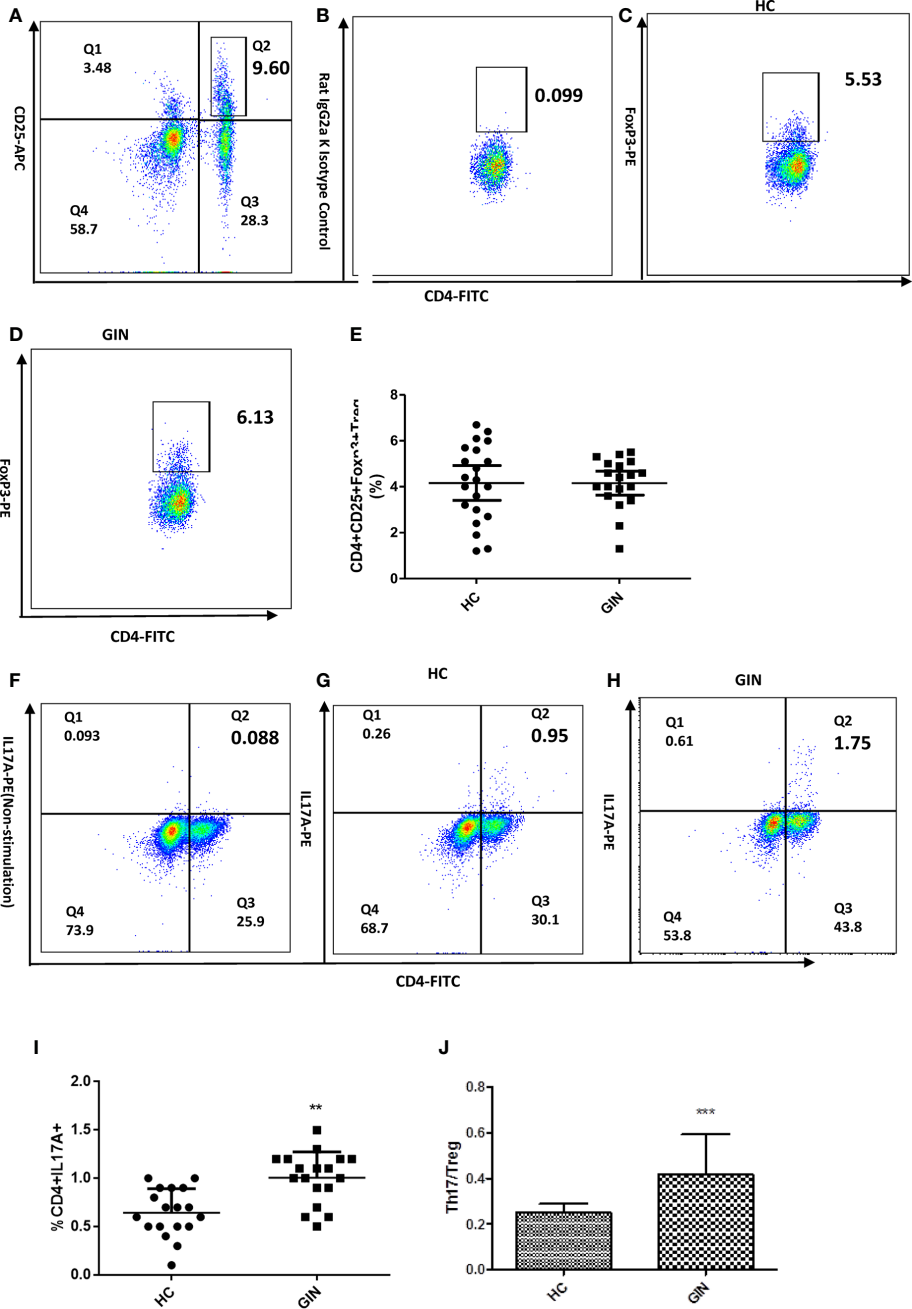
Proportions of CD4+CD25+FoxP3+ Treg and CD4+IL-17+ Th17 cells in gingivitis patients and healthy controls. **(A)** Dot plot int the upperright quadrant represents CD4+ CD25 + T Cells. **(B)** Isotype control staining of FoxP3. **(C)** Dot plot represents Treg cells from a healthy control subject **(D)** Treg cells from a representative patient with gingivitis. **(E)** CD4+CD25+Foxp3+Treg percentages. **(F)** Non stimulation of Leukocyte Activation Cocktail of the Th17 cells in a healthy control**.(G)** Dot plot in the upper right quadrant represents Th17 cells from a healthy control subject. **(H)** Th17 cells from a representative patient with gingivitis. **(I)** CD4+IL-17+Th17 percentages. **(J)** the ratio of Th17/Tre. Data presented are means ± SD. **P < 0.01, ***P < 0.001 *versus* healthy control.

Flow cytometry revealed significantly higher percentage of CD4^+^IL-17A^+^ T cells in the gingivitis group, relative to healthy controls (**Figures 2F–I**). With regards to the proportion of Treg cells, we found no significant differences in percentage of CD4^+^ CD25^+^Foxp3^+^ Treg cells between the groups (**Figures 2A–E**). Analysis of CD4^+^IL-17A^+^Th17/CD4^+^CD25^+^Foxp3^+^Treg cells revealed a higher ratio in the gingivitis group, relative to healthy controls (**Figure 2J**).

### Expression Patterns of TGF-β, IL-17A, Foxp3 and RORγt

**Figure 3:**
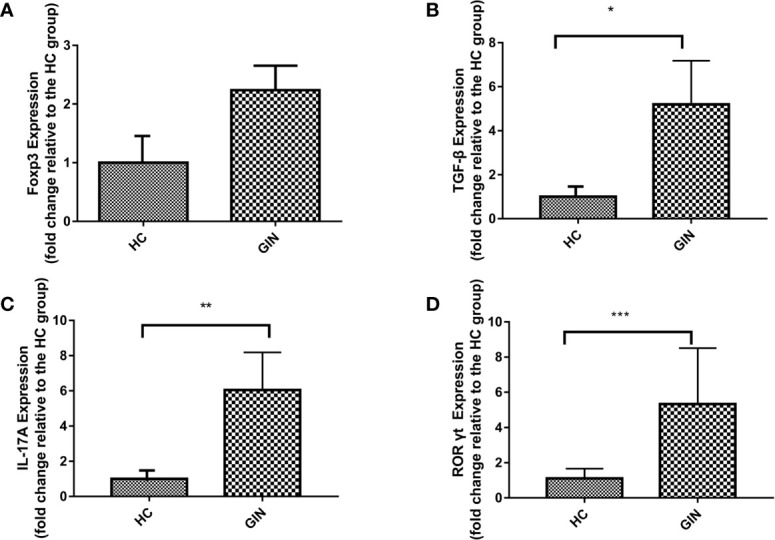
Expression patterns of Foxp3, TGF-β, IL-17A, RORγt in gingivitis patients and healthy controls. Data presented are means±SD. *P < 0.05, **P < 0.01 versus healthy control. ***P < 0.001 versus healthy control. **(A)** Expression patterns of Foxp3 in gingivitis patients and healthy controls. **(B)** Expression patterns of TGF-β in gingivitis patients and healthy controls. **(C)** Expression patterns IL-17A of in gingivitis patients and healthy controls. **(D)** Expression patterns of RORγt in gingivitis patients and healthy controls.

We used qRT-PCR to analyze expression of Th17-related genes. Results revealed significant upregulation of IL-17A and TGF-β in gingivitis patients, relative to healthy controls (**Figures 3B, C**). Similarly, specific transcription factors Foxp3 (of Treg) and RORγt (of Th17) were upregulated in gingivitis patients, relative to healthy controls, although only RORγt was statistically different between the group (**Figures 3A, D**).

### Levels of TGF-β, IL-4, IL-6, IL-10 and IL-17A in the Plasma

**Figure 4:**
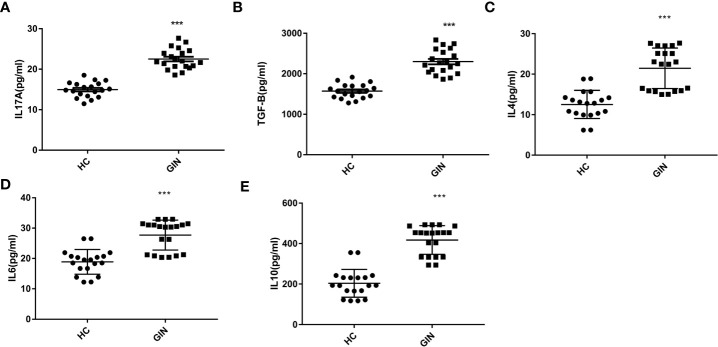
**(A)** Levels of IL-17A in the plasma of gingivitis patients and healthy controls. **(B)** Levels of TGF-β in the plasma of gingivitis patients and healthy controls. **(C)** Levels of IL-4 in the plasma of gingivitis patients and healthy controls. **(D)** Levels of IL-6 in the plasma of gingivitis patients and healthy controls. **(E)** Levels of IL-10 in the plasma of gingivitis patients and healthy controls. ***P < 0.001 versus healthy control.

We explored levels of Th17/Treg-related cytokines, targeting TGF-β, IL-4, IL-6, IL-10 and IL-17, using ELISA, and found that all cytokines were significantly elevated in the gingivitis patients, relative to healthy controls (**Table 3** and **Figure 4**).

**Table 3:** Levels of IL-17A, IL-4, IL-6, IL-10 and TGF-β in patients’ plasma.

Groups	number	IL-17A (pg/ml)	TGF-β (pg/ml)	IL-4 (pg/ml)	IL-6 (pg/ml)	IL-10 (pg/ml)
HC	19	14.97 ± 1.81	1572.63 ± 173.93	12.55 ± 0.80	18.89 ± 0.93	204.10 ± 15.75
GIN	20	22.52 ± 2.48***	2300.6 ± 291.47***	21.46 ± 1.12***	27.71 ± 1.10***	417.90 ± 15.84***

***P < 0.001 versus healthy control.

### Levels of SIgA in Saliva

**Figure 5:**
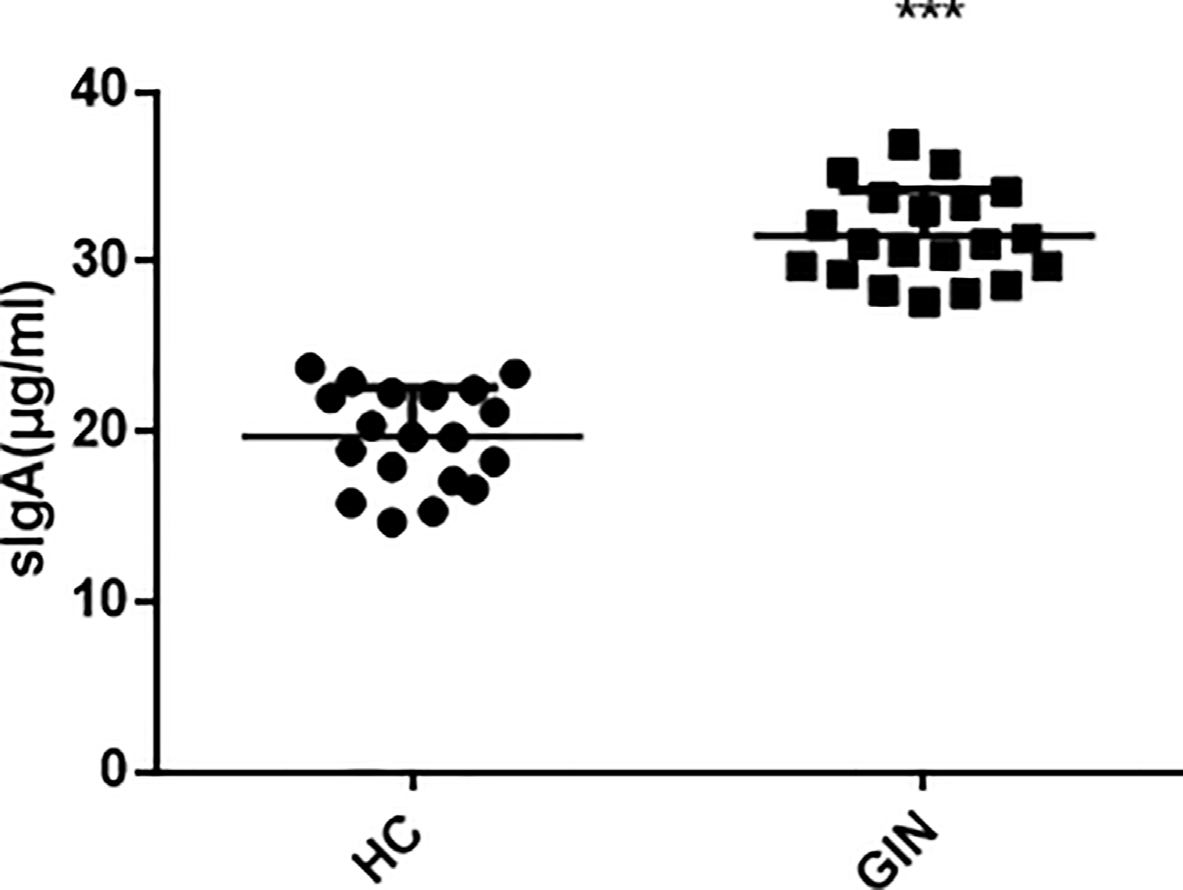
Levels of SIgA in patients’ saliva. ***P < 0.001 *versus* healthy control.

To explore the condition of oral local immunity, we analyzed levels of SIgA in saliva, using ELISA, and found significantly higher levels in the gingivitis (31.52 ± 2.70 μg/ml), relative to the healthy control (19.74 ± 2.88 μg/ml) group (**Figure 5**).

### Analysis of Metabolic Profiling From the Gingivitis and Healthy Control Groups

**Figure 6:**
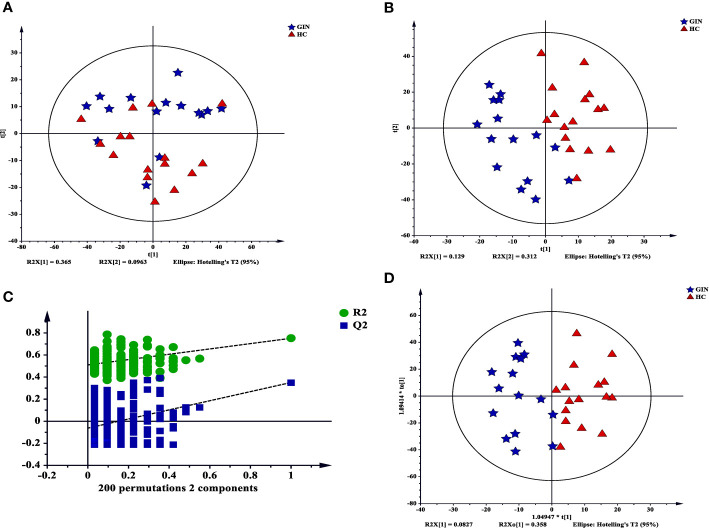
Multivariate statistical analysis for GC-MS based on metabolic profiling. **(A)** a PCA score plot data from the healthy control group (red) the *versus* the gingivitis group (blue); **(B)** a PLS-DA scores plot data fron the healthy control group (red) *versus* the gingivitis group (blue); **(C)** internal cross validation plot with a permutation test repeated 200 times; **(D)** OPLS-DA scores plot data from the healthy control group (red) *versus* the gingivitis group (blue).

We used principal component analysis plot to analyze major sources of metabolic differences among palma samples. We found a clear separation between the groups, suggesting that gingivitis patients may exhibit a specific metabolic pattern (R^2^X = 0.666, Q^2^ = 0.427) (**Figure 6A**). To verify these metabolic differences, we adopted the PLS-DA model under a supervised analysis. Results revealed cumulative R^2^X, R^2^Y and Q^2^ of 0.441, 0.752, and 0.351, respectively, affirming the model’s predictive power (**Figure 6B**). Similarly, **Figure 6C** revealed that the data in this PLS-DA model were reliable, thus can be used for further analysis. To maximize class discrimination and identify gingivitis-related metabolites, we constructed the OPLS-DA model (R^2^X = 0.441, R^2^Y = 0.752, Q^2^ = 0.419) (**Figure 6D**). The resulting validation plots indicated successful establishment of an accurate model.

**Figure 7:**
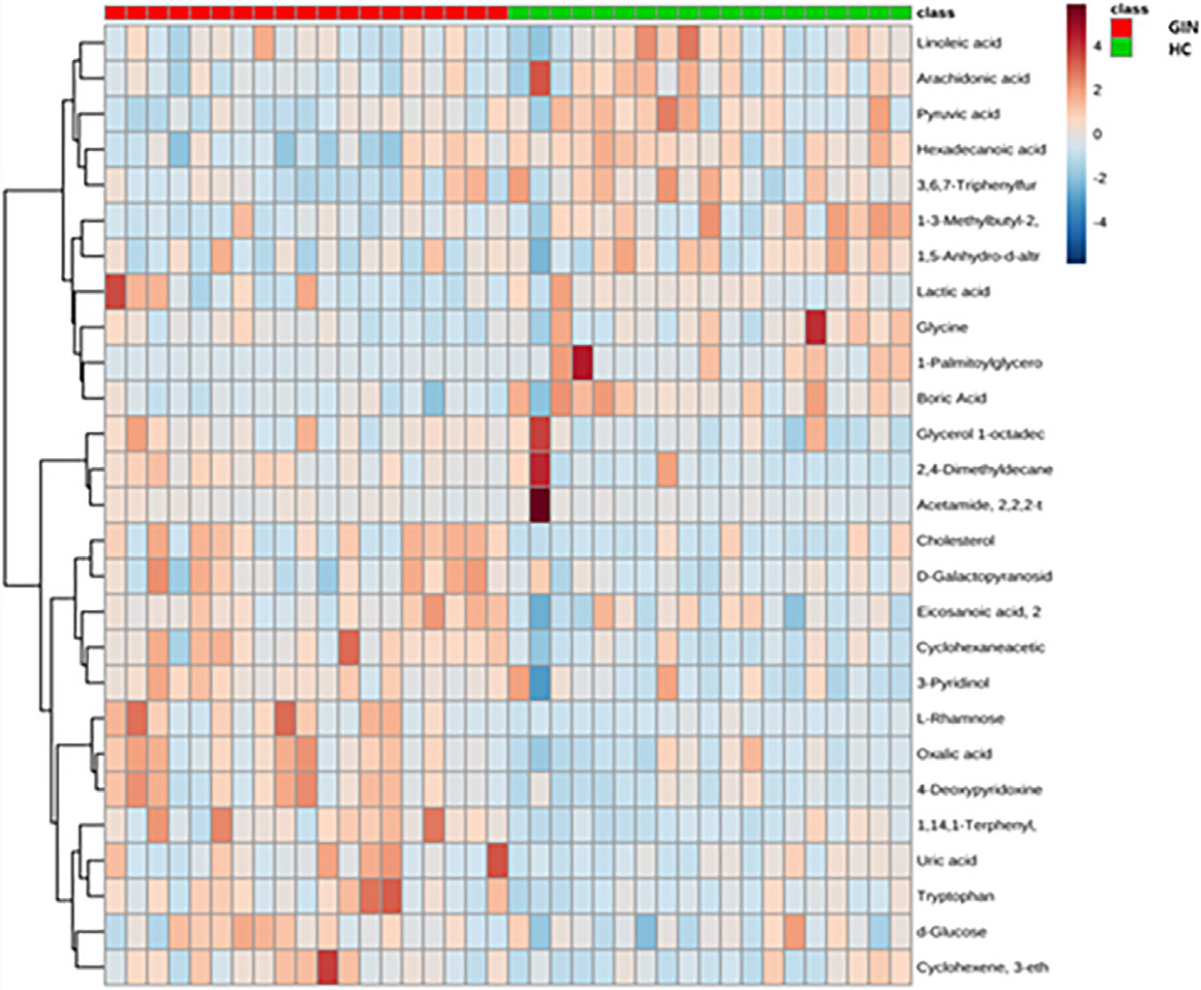
Cluster analysis graph of differential metabolites between gingivitis group (red) and healthy control group (blue).

We then applied a multidimensional analysis method, OPLS-DA (VIP > 1, *P* < 0.05), to search for potential metabolites potentially causing the differences between groups, and identified 18 significantly different metabolites between the gingivitis and healthy control groups. Eight of these metabolites, namely cholesterol, glycerol 1-octadecanoate, d-glucose, uric acid, cyclohexaneacetic acid, 3-pyridinol, tryptophan, and 2,4-dimethyldecane increased from high to low in the gingivitis compared with the control group. Similarly, cluster relationship revealed that the remaining 10 metabolites, namely, lactic acid, glycine, linoleic acid, 1-palmitoylglycerol, hexadecanoic acid, arachidonic acid, pyruvic acid, 1-(3-Methylbutyl)-2,3,4,6-tetramethylbenzene, 1,5-anhydro-d-altritol, and boric acid had higher levels in gingivitis patients that healthy controls (**Figure 7**).

**Figure 8:**
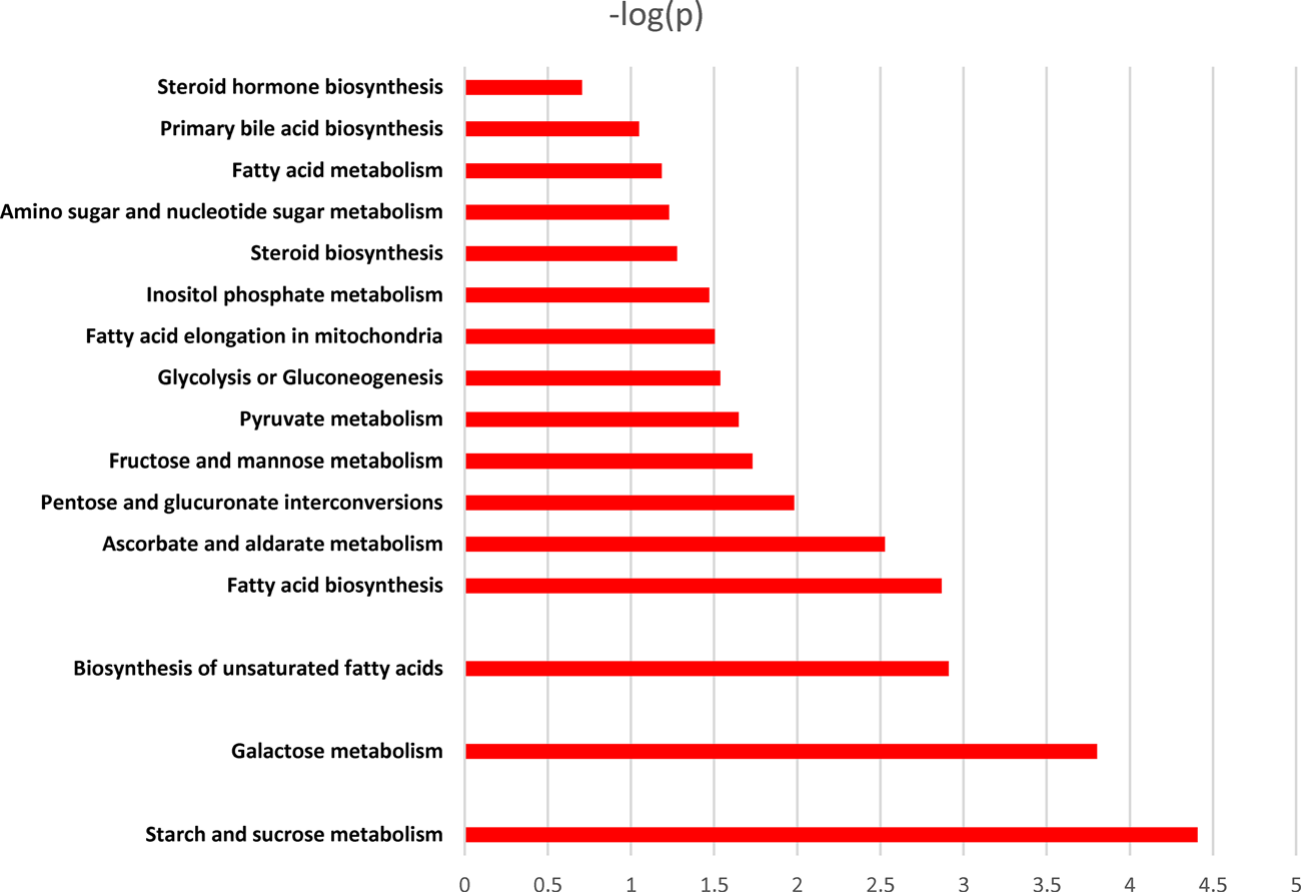
Pathway enrichment analysis of different metabolites. X-axis: log 10 (P value); Y-axis: metabolic pathways.

Pathway enrichment analysis of the differentially expressed metabolites revealed that they were predominantly enriched in the biosynthesis of unsaturated fatty acids, pyruvate metabolism, glycolysis or gluconeogenesis, glycine, serine and threonine metabolism, cyanoamino acid metabolism, and linoleic acid metabolism, among others (**Figure 8**).

**Figure 9:**
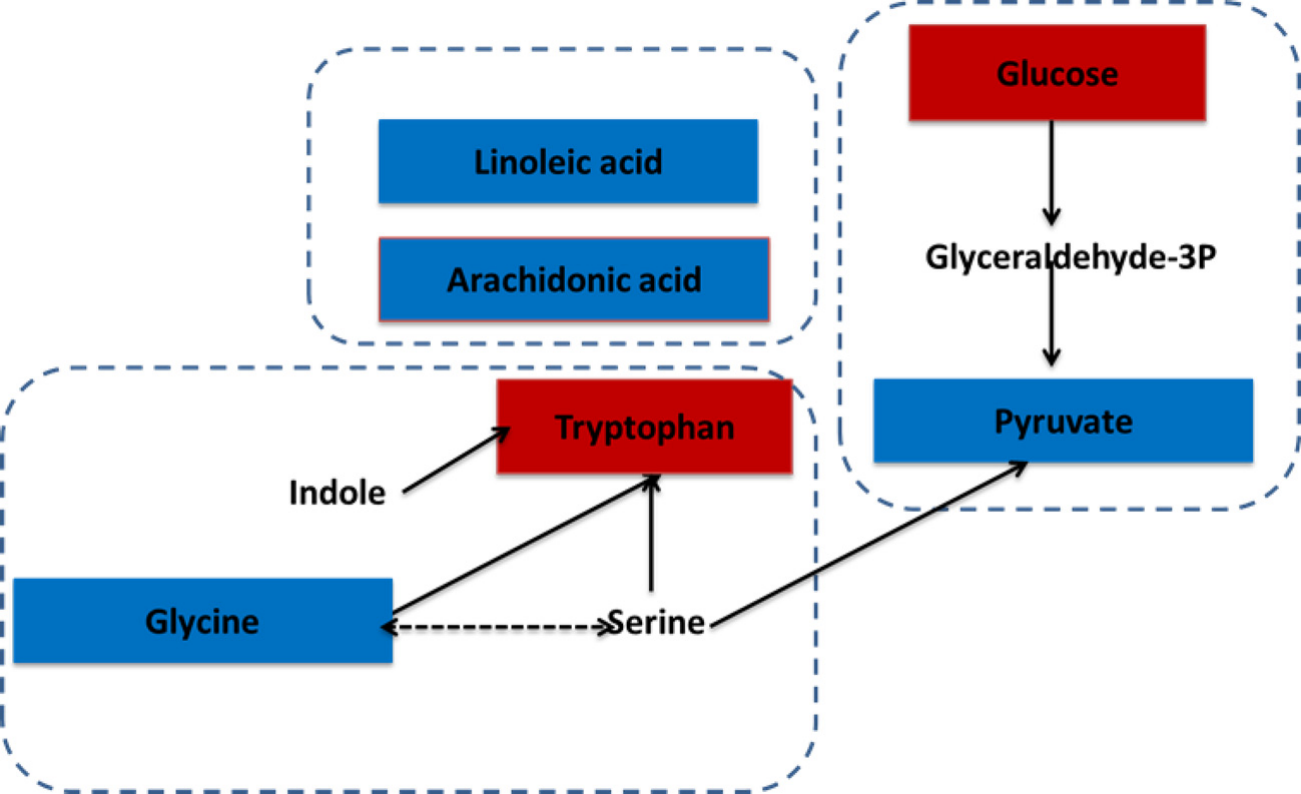
The correlation between differential metabolites and gingivitis. The metabolites, which are marked red, indicating significantly increased in the gingivitis group. The blue marked metabolite indicates a decrease.

A metabolic network of the differentially metabolites, targeting glycolysis, fatty acid metabolism, and tryptophan metabolism revealed substantial increase of glucose and tryptophan, whereas linoleic acid, arachidonic acid, glycine, and pyruvate were decreased in the gingivitis group (**Figure 9**).

### Ingenuity Pathway Analysis

**Figure 10:**
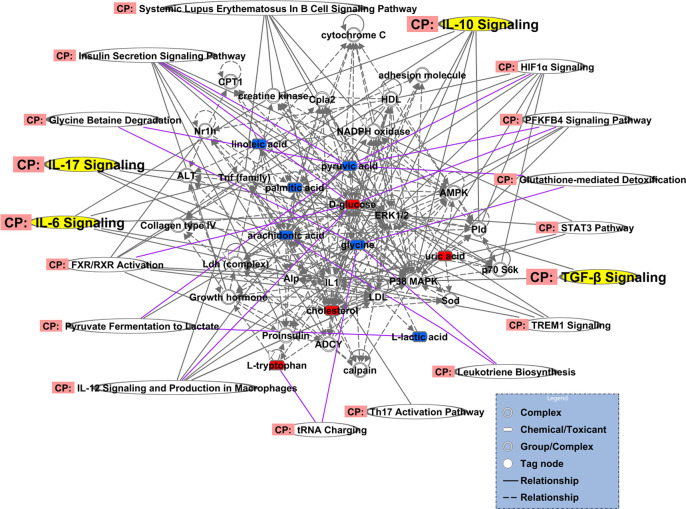
Differential expression network of plasma metabolites in gingivitis patients. The metabolites, which are marked in red, indicating significantly increased in gingivitis group. The blue marked metabolite indicates a decrease.

The 18 regulated metabolites were imported into IPA software for biological pathway analysis. At last, we conducted differential expression network of plasma with 10 different metabolites. The results of IPA analysis showed that influenced the IL-17 signaling, TGF-β signaling, IL-6 signaling, IL-10 signaling and so on(**Figure 10**). These different metabolites mainly correlated with ERK1/2, P38MAPK, IL1, Tnf (family) and other molecules in these pathways.

## Discussion

Regulating the balance of Th17/Treg cells plays a pivotal role in controlling development of autoimmune and certain inflammatory diseases, because the signaling pathways for Th17 and Treg cells are similar. Previous studies identified an association between CD4^+^CD25^+^Treg and periodontitis. For example, Nakajima et al. (15) observed CD4^+^CD25^+^Treg in all gingivitis, and periodontitis patients alongside healthy individuals, based on immunohistochemical staining of clinical gum specimens, although the proportion was higher in periodontitis patients. In another study, Ernst et al. (16) reported occurrence of some CD4^+^CD25^+^Treg in bone resorption lesions during periodontal bone destruction. Their results revealed a significantly lower site and this was negatively correlated with destruction of the periodontal tissue. Collectively, these studies suggest that the number of CD4^+^CD25^+^Treg may change during the pathological process of gingivitis and periodontitis. Similarly, Ohlrich et al. (5) revealed that autoimmune response caused by Th17/Treg imbalance is one of the crucial factors that cause periodontitis. Recently, Figueredo et al. (17) demonstrated that activation of distinct T and B cell subtypes is a decisive phenomenon in defining whether an inflammatory lesion will stabilize as chronic gingivitis or will progress as a tissue destructive periodontitis.

In recent years, numerous studies have described existence of a mutually antagonistic and balanced relationship between Th17 and Treg cells with regards to differentiation and function. The possible mechanism is that TCR stimulates initial CD4^+^T cells, and TGF-β alone can induce the expression of Foxp3 and RORγt (18). Therefore, TGF-β is a common determinant for Th17 and Treg cells, primarily acting to induce co-expression of related transcription factors RORγt and Foxp3. Presence of proinflammatory cytokines, such as IL-6 or IL-21, mediates activation of the STAT3 pathway to inhibit expression of Foxp3, thereby inhibiting differentiation of Treg cells, whereas upregulation of RORγt promotes differentiation of Th17 cells (19). McGeachy et al. (20) showed that Th17 cells could also produce IL-10, and this was accompanied by up-regulation of RORγt and production of IL-17. It is generally believed that IL-10 is secreted by Treg cells and it exerts immunosuppressive characteristics as well as anti-inflammatory activity. In fact, IL-17^+^IL-10^+^ cells have been found to function like Th17 and Treg cells. *In vivo* studies, using mouse models, have shown that Foxp3 ^+^ RORγt ^+^ T cells have the ability to produce IL-17 (21). Moreover, CD4 ^+^ Foxp3 ^+^ Treg cells are also present in human peripheral blood and lymph tissues, which can express CCR6 and have the ability to produce IL-17. Such IL-17-producing Treg cells co-express Foxp3 and RORγt (22).

Results of the present study revealed significant upregulation of TGF-β and IL-17A in gingivitis patients, relative to healthy controls. Similarly, Foxp3 and RORγt also exhibited an increasing growing trend in the gingivitis group. ELISA results revealed higher levels of IL-17A, IL-10, IL-6 and TGF-β in plasma of gingivitis patients relative to normal controls. Due to the increase of TGF-β and FoxP3, the Treg cells, in general, should increase. However, we found CD4^+^CD25^+^Foxp3^+^Treg cells no significant difference compared with the healthy control group. Liang Zhou et al. claimed that the decision of antigen-stimulated cells to differentiate into Th17 or Treg cells depends upon the cytokine-regulated balance of RORγt and Foxp3 (21).In addition, IL-6 induces the generation of Th17 cells from naïve T cells together with TGF-β and inhibits TGF-β-induced Treg (iTreg) differentiation (23). In our study, the ratio of RORγt/Foxp3 in GIN group was significantly higher than that in HC group(*P*<0.01). Therefore, the increase of TGF-β along with the higher levels of RORγt, accompanied by IL-6 in peripheral blood lymphocytes, differentiate into Th17 cells. Moreover, another possible cause may be the transformation of Treg into Th17 cells.

Taken together, a possible mechanism of gingivitis is that an increase in TGF-β content, coupled with IL-10 upregulation, in peripheral blood lymphocytes activated Treg cells, while transcription factor Foxp3 participated in a synergistic effect, thereby producing CD4^+^CD25^+^Foxp3^+^Treg cells. However, we found CD4^+^CD25^+^Foxp3^+^Treg cells no significant difference compared with the healthy control group, possibly due to transformation of Treg into Th17 cells. The high TGF-β levels, accompanied by IL-6 in peripheral blood lymphocytes, activated Th17 cells and caused elevated percentage of CD4^+^ IL-17A^+^ Th17 cells in the peripheral blood of gingivitis patients, thereby secreting corresponding inflammatory factor IL-17A. This caused local inflammation to occur in gingivitis patients which consequently stimulated local immunity. Analysis of Th17/Treg cell percentage ratio revealed significantly higher cell numbers in gingivitis patients than in normal controls, further suggesting that Th17/Treg imbalance may be the underlying mechanism of gingivitis.

Secretory immunoglobulin A (SIgA) is a major human mucosal immune antibody, that is predominantly present in external secretions, such as saliva, tears, milk, gastric, intestinal, respiratory, and urogenital tract fluids. SIgA, with a molecular weight of about 400 kD, is composed of double IgA, J chain and secretion piece (sc) covalently. J chain and IgA (d) are produced by locally activated B cells, sc is synthesized by mucosal epithelial cells, awhile sc covalently binds with J chain to form a dimer structure with IgA (d), namely SIgA (24, 25). The mechanisms of SIgA action mainly involve inhibition of microbial adhesion to the mucosal surface, neutralization of toxins and sterilization, as well as anti-inflammatory regulation and conditioning. Previous studies have shown that SIgA is closely correlated with occurrence and development of many diseases, such as chronic sinusitis (26), repeated respiratory tract infection (27), and oral cancer (28), among others. Regulating sIgA levels is beneficial in improving mucosal immunity of clinical diseases.

Recent studies have shown that SIgA is significantly elevated in mixed saliva of gingivitis patients relative to healthy controls (29). Other evidences have reported that once the epithelial barrier has been penetrated, the underlying connective tissue’s defense is primarily an inflammatory response. Immunoglobulins primarily act to provide antibodies to enhance phagocytosis and promote host defense mechanisms at the site of infection (30). Some researchers have described SIgA’s role in intestinal immunity, although it may bind to T cells and play another local immunomotor role, thereby regulating production of cytokines, such as IL-4, IL-10 and TGF-β in mucosa. Therefore, entry of SIgA into mucosa through Peyerpatch lymph nodes (PP) may play an important immunomodulatory role in protecting the integrity of the intestinal barrier (31). Mucosal-associated lymphoid tissue (MALT), which comprises both PP in intestinal tract as well as oral and gingival mucosa, represents an important defense barrier in the human body.

Results of the present study revealed significantly higher levels of SIgA in saliva as well as TGF-β, IL-4 and IL-10 in plasma of gingivitis patients, relative to healthy controls, suggesting enhanced local humoral immunity. The possible mechanism for this phenomenon could be due to stimulated inflammation which mediated secretion of SIgA in the oral. Later, SIgA worked with lysozyme and complement in saliva to dissolve local bacteria and toxins in the gums. In addition, part of SIgA may combine with T cells in the body, and as a result, cause secretion of cytokines, such as TGF-β, IL-4 and IL-10 to strengthen local immune function and further mobilize the body’s own immune response to gingivitis.

Previous studies have shown that periodontitis patients exhibit different metabolites in their saliva, serum, and gingival crevicular fluid, which may help unravel the underlying molecular mechanism of periodontal disease (32, 33). In this present investigation, gingivitis patients exhibited abnormal amino acid metabolism, as evidenced by down-regulated glycine and up-regulated tryptophan in plasma. According to a previous study, glycine can augment production of anti-inflammatory cytokine IL-10, and suppress synthesis of proinflammatory cytokines IL-1β and TNF-α by LPS-activated monocytes (34). Air polishing, using a conventional air-polishing device with fine-grain (DV90: 63 mm) glycine powder, has been shown to efficiently and safely remove the subgingival biofilm in periodontal pockets, aimed directly into the periodontal pocket (35). Tryptophan (Trp) is one of the least abundant and biosynthetically energy-intensive essential amino acids, necessary for protein synthesis. The major catabolic route for Trp in mammals is the kynurenine (Kyn) pathway, which accounts for > 90% of all peripheral Trp metabolism (36). Moreover, Trp is constitutively oxidized by tryptophan 2, 3-dioxygenase in liver cells, while in other cell types, it is catalyzed by an alternative inducible indoleamine-pyrrole 2, 3-dioxygenase (IDO) under certain pathophysiological conditions. IDO can induce production of Treg cells, which represents an important regulator that exerts immune regulation and maintains immune homeostasis. Interestingly, intravenous injection of IDO inducer ISS-ODN into IL-6 knockout rats caused an increase in Treg levels in their spleen, while subcutaneous injection of IDO1 inhibitor 1-MT activated IL-6 expression in spleen pDC, thereby transforming Treg into Th17 cells (37). Results of the present study revealed the significant upregulation of Trp in gingivitis patients, implying that the kynurenine (Kyn) pathway was downregulated. Therefore, IDO activity decreased which led to an increase in the ratio of Th17 cells in the gingivitis group.

Moreover, we observed abnormal fatty acid metabolism in gingivitis patients, as evidenced by down-regulated unsaturated fatty acids, linoleic acid, and arachidonic acid in plasma. Linoleic acid (LA) can be converted to the metabolically active arachidonic acid (AA), which plays a critical role in inducing inflammation and adipogenesis as well as endocannabinoid system regulation AA, generated from LA or dietary. In addition, arachidonic acid intake can be converted into numerous inflammatory metabolites by cytochrome P450, cyclooxygenase, and lipoxygenase pathways (38). Diabetes mellitus is a significant risk factor for periodontal disease, and has been shown to predispose patients to severe periodontal inflammation. Linoleic acid is also available as a dietary supplement, and has been implicated in weight loss. Moreover, previous studies have shown that addition of commercial conjugated linoleic acid (CLA) products to the diet can significantly alleviate alveolar bone loss, enhance osteoblastic activity, and lower osteoclastic activity in the diabetic Wistar rats (39). Furthermore, fatty acid metabolism is essential for T cells, where it can regulate the balance between effector T cells and Tregs. Fatty acid synthesis is the key to promoting T and B cell functions. Specifically, fatty acid and sterol synthesis are essential raw materials for these cells’ proliferation. In fact, fatty acid synthesis promotes Th17 cell differentiation, while the type of intracellular synthesis s closely associated with the type of cytokine produced by effector T cells.

Glucose was significantly upregulated in the plasma of gingivitis patients, whereas pyruvate was down-regulated. Pyruvate is a central intermediate of sugar metabolism in the body, that helps determine mutual transformation of various substances in the body. Down-regulation of pyruvate in patients with gingivitis may be ascribed to the increased consumption of pyruvate, and this causes accumulation of glucose in the body, indicating abnormal metabolism of glycolysis. On the other hand, lactic acid may be produced through the gluconeogenesis pathway to produce glucose and glycogen, thereby down-regulating lactic acid in gingivitis patients. Activated CD4^+^T cells (such as Th17 cells) and CD8^+^CTL primarily obtain energy through aerobic glycolysis and glutamine cleavage pathways. At the same time, these rapidly dividing cells also synthesize a large number of esters. However, Treg and memory CD8^+^T cells are similar to the initial T cells, in that they don’t depend on glycolysis but mainly rely on ester oxidation for energy provision. In the present study, it is possible that Th17 cells increased by obtaining energy from glycolysis.

Through the IPA analysis, we found that these different metabolites especially D-glucose, Glycine and Pyruvic acid were closely related IL-17 signaling, TGF-B signaling, IL-6 signaling pathway, and IL-10 signaling pathway which play significant role in Th17/Treg.

**Figure 11:**
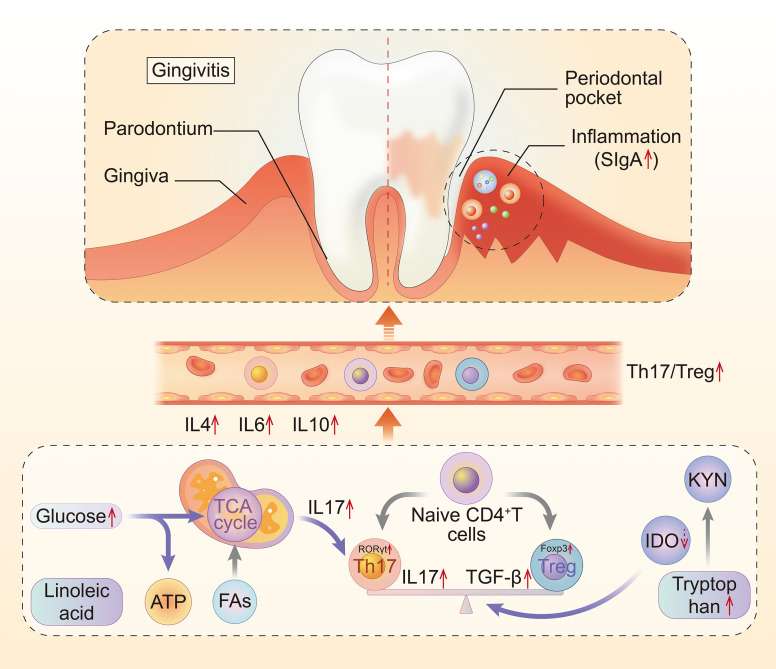
The mechanism of the metabolic disturbance and Th17/Treg imbalance in gingivitis. Th17, T helper cell 17; Treg, regulatory T cell; Fas, fatty acid metabolism; IDO, indoleamine-pyrrole 2,3-dioxygenase; KYN, kynurenine.

The underlying mechanism of local inflammation and systemic injury in gingivitis is often complex, with Th17/Treg playing an important role in progression of inflammation. The present study uncovered the role played by Th17/Treg imbalance and immune metabolism in development of gingivitis. Taken together, our findings indicate that impaired glycolysis, as well as fatty and amino acid metabolism in the plasma of gingivitis patients interact with Th17/Treg imbalance in the body (**Figure 11**).

## Conclusion

In summary, our results reveal that Th17 and Treg cells are significantly elevated in gingivitis patients. Disturbance to immunometabolism might serve as a potential therapeutic target for development of treatment therapies for systemic diseases.

## Data Availability Statement

The original contributions presented in the study are included in the article/**Supplementary Material**. Further inquiries can be directed to the corresponding authors.

## Ethics Statement

The studies involving human participants were reviewed and approved by the Ethics Committees of Zhejiang Chinese Medical University. The patients/participants provided their written informed consent to participate in this study.

## Author Contributions

WW and XW contributed equally to this paper. WW contributed to the conception of the study. WW, XW, HL, GX, and YD performed experiments. SL and TZ collected the data. LX organized the methodology. YF supervised the research. WW wrote the first draft of the manuscript. All authors contributed to the article and approved the submitted version.

## Funding

This research was supported by the National key basic research development plan (973 Plan) project (NO. 2014CB543001); National Natural Science Foundation of China (NO.82004238; 81673857); Natural Science Foundation of Zhejiang Province (NO.LQ20H270007; LBY21H270001) and Zhejiang Medicine and Health Science and Technology Project (NO. 2021KY843).

## Conflict of Interest

The authors declare that the research was conducted in the absence of any commercial or financial relationships that could be construed as a potential conflict of interest.
