# Supplementary material for: Metabolic Disturbance and Th17/Treg Imbalance Are Associated With Progression of Gingivitis
Source: Front Immunol. 2021 Jun 21;12:670178. doi: 10.3389/fimmu.2021.670178 (PMC8257051; doi:10.3389/fimmu.2021.670178)
Supplement: Supplementary file 1 [file DataSheet_1.pdf]

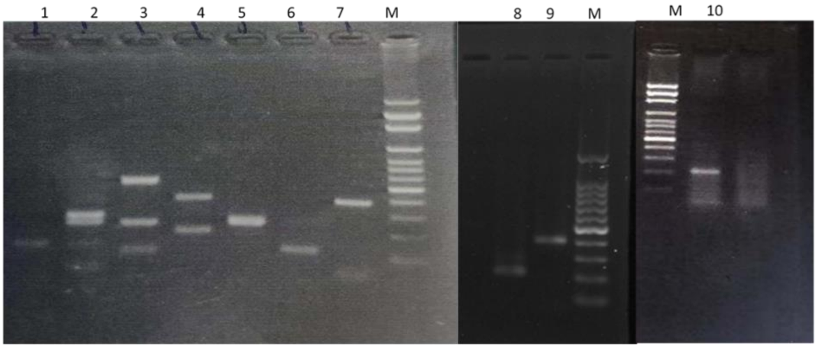

FIGURE S1 | The electrophoresis identification picture of the amplified product of qRT-PCR.M: marker. 1: GAPDH-1.2-4:The products amplified by the primers have non-specific bands, which were not included in the experiment.5:Foxp3-1.6:TGF-β.7: Foxp3-2.8:IL-17A.9: GAPDH-2.10: RORC.

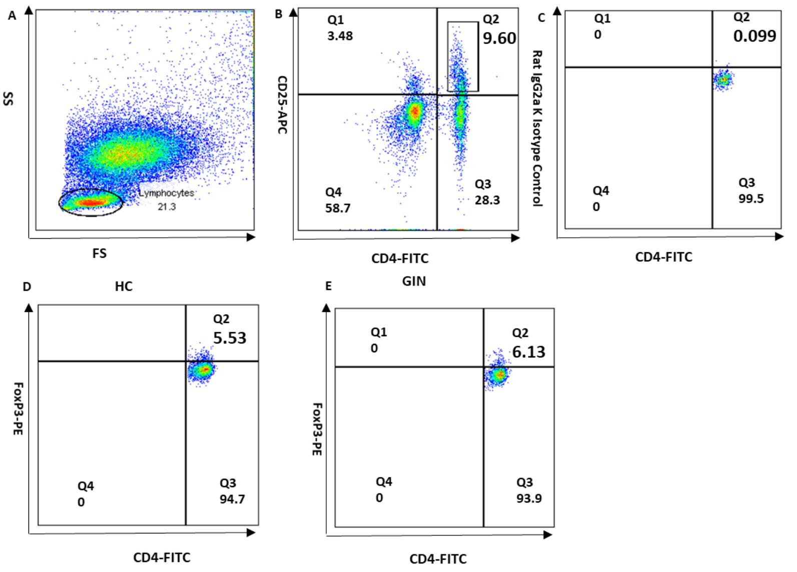

FIGURE S2 | Proportions of CD4+CD25+FoxP3+ Treg cells in isotype control. A:Lymphocyte in Treg cells detection. B: Dot plot in the upper right quadrant(Q2) represents CD4+ CD25 + T Cells. C:Isotype control staining of FoxP3, Dot plot in the upper right quadrant (Q2) represents CD4+CD25+FoxP3+ Treg cells. D. Dot plot in the upper right quadrant represents Treg cells from a healthy control subject .E: Treg cells from a representative patient with gingivitis.

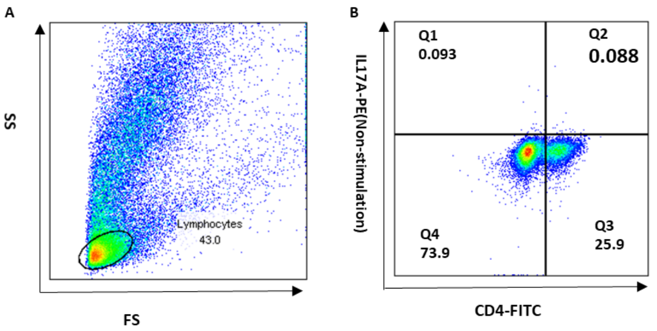

FIGURE S3 | Proportions of CD4+IL-17A+ Th17 cells in biological control. A:Lymphocyte in Th17 cells detection. B:Non-stimulation of the Leukocyte Activation Cocktail of the Th17 cells from one healthy control subject, Dot plot in the upper right quadrant (Q2) represents CD4+IL-17A+ Th17 cells
